# Job Stress, Burnout and Coping in Police Officers: Relationships and Psychometric Properties of the Organizational Police Stress Questionnaire

**DOI:** 10.3390/ijerph17186718

**Published:** 2020-09-15

**Authors:** Cristina Queirós, Fernando Passos, Ana Bártolo, Sara Faria, Sílvia Monteiro Fonseca, António José Marques, Carlos F. Silva, Anabela Pereira

**Affiliations:** 1 Faculty of Psychology and Education Sciences, University of Porto, 4200-135 Porto, Portugal; up201403461@fpce.up.pt (S.F.); mipsi11157@fpce.up.pt (S.M.F.); 2 Psychology Unit of the Portuguese National Police, 2605-000 Belas-Sintra, Portugal; fmpassos@psp.pt; 3 Center for Health Technology and Services Research (CINTESIS), Department of Education and Psychology, University of Aveiro, 3810-193 Aveiro, Portugal; anabartolo@ua.pt; 4 School of Health, Polytechnic of Porto, 4200-072 Porto, Portugal; ajmarques@ess.ipp.pt; 5 Department of Education and Psychology, University of Aveiro, 3810-193 Aveiro, Portugal; csilva@ua.pt (C.F.S.); anabelapereira@ua.pt (A.P.)

**Keywords:** job stress, organizational stress, burnout, coping, police officers, questionnaire validation

## Abstract

Policing is a stressful occupation, which impairs police officers’ physical/mental health and elicits burnout, aggressive behaviors and suicide. Resilience and coping facilitate the management of job stress policing, which can be operational or organizational. All these constructs are associated, and they must be assessed by instruments sensitive to policing idiosyncrasies. This study aims to identify operational and organizational stress, burnout, resilient coping and coping strategies among police officers, as well to analyze the psychometric properties of a Portuguese version of the Organizational Police Stress Questionnaire. A cross-sectional study, with online questionnaires, collected data of 1131 police officers. With principal components and confirmatory factor analysis, PSQ-org revealed adequate psychometric properties, despite the exclusion of four items, and revealed a structure with two factors (poor management and lack of resources, and responsibilities and burden). Considering cut-off points, 88.4% police officers presented high operational stress, 87.2% high organizational stress, 10.9% critical values for burnout and 53.8% low resilient coping, preferring task-orientated than emotion and avoidance coping. Some differences were found according to gender, age and job experience. Job stress and burnout correlated negatively with resilient coping, enthusiasm towards job and task-orientated coping. Results reinforce the importance to invest on police officers’ occupational health.

## 1. Introduction

Nowadays, policing is considered a stressful professional occupation [1,2,3,4] requiring police officers to cope with danger, uncertainty and unpredictability. Thus, their job stress is increasing, leading to burnout, mental/psychological disorders or even police suicide [4,5,6,7,8], forcing police officers to be resilient and to develop coping strategies to face all job demands.

Despite policing stress sources being multiple [4,9], they can be divided in operational and organizational stressors, being operational and organizational stress two distinct and specific constructs for police forces [10]. According to several authors [4,10,11,12,13,14,15], operational stressors are related with policing specificities such as working in shifts, danger and risk of injury, critical incidents, traumatic events, citizen criticism of police officers’ behavior, perception of policing by society, fear of excessive use of force, aggressive interactions, work–family conflict, etc. On the other hand, organizational stressors are related with Police as an institution/organization, including stressors such as conflicts with supervisors/colleagues, lack of material/human resources, work overload, excessive administrative tasks, leadership problems, etc., as well each police department/command/police station specific way of functioning.

When job stress becomes chronic, it strongly affects physical and mental health [16,17], and nowadays stress is considered as a psychosocial risk at work [18,19]. When the response to chronic job stress is inadequate, burnout appears as an occupational phenomenon [20,21] defined as a “prolonged response to chronic emotional and interpersonal stressors on the job, [expressed on] three dimensions of exhaustion, cynicism and inefficacy” [22] (p. 397). Regarding police officers’ burnout, innumerous studies found high levels of exhaustion and depersonalization, relationships between burnout and mental/psychological problems such as anxiety, depression and post-traumatic stress disorder [4,23,24,25,26], as well as depersonalization as a coping strategy, emotional suppression and difficulties to express true emotions [27]. Furthermore, police officers’ burnout can be related with internal or external aggression, a current social and political concern. Regarding external aggression, excessive use of force among police forces can be a result of high levels of stress [2,13,16,28,29,30], which can make police officers assess the situations as more threatening than what they really are [31]. Regarding internal aggression, several studies [7,8,32,33] alerted for suicide among police officers due to the easy access to a gun, due to situations which elicit post-traumatic stress disorder, stressful conditions during policing, depression and burnout. Heyman et al. [34] (p. 7) concluded “police officers (…) are more likely to die by suicide than in the line of duty”.

Considering policing hazards and stressful working conditions, police officers need to be resilient and to use adequate coping strategies to manage stress, burnout and psychological consequences of critical incidents. Resilience can be viewed as a process of positive adaptation during and after a significant adversity, as well as a stress-coping ability allowing to deal with the adverse situation and to recover and grow after that [35,36]. Coping consists of cognitive and behavioral efforts to manage/cope/reduce stress elicited by significant events, using problem-focused or emotion-focused strategies [37]. Problem-focused strategies try to modify stress sources through problem solving, decision-making, confrontation, social support seeking and/or direct action. Emotion-focused strategies try to regulate/reduce/manage distressing emotions by modifying the cognitive meaning of the stressful situation, without changing the situation itself, seeking emotional support or using self-control, distancing, positive appraisal, acceptance and/or avoidance. Resilient persons frequently use more problem-focused strategies than emotion/avoidance-focused strategies since resilient coping implies a positive adaptation in stressful situations, combining the longitudinal process of resilience with the specific moment were a coping strategy is used to solve the stressful situation [38,39]. Research about resilience and coping among police officers showed that coping strategies are related with job stress [40,41,42], and police officers preferred to use problem-focused strategies [43,44,45], which increased with job experience [40] and are related with resilience [46,47]. Moreover, avoidance strategies are related with substance/alcohol use to cope with stressful situations [48,49], while emotion-focused strategies are related with depression, anxiety and poor mental health [50], as well with burnout [51].

Research about stress, burnout and coping refers to the influence of individual characteristics. Gender and age are the most frequently examined, showing that women experienced more emotional exhaustion, less depersonalization [52] and more stress; they preferred to use emotion-focused strategies [53] and seemed to be more resilient [54] than men. Older people also showed to be more resilient [54]. Among police officers, some studies found no differences [3,55], while others found that women experience more burnout [56], more emotional exhaustion and less depersonalization [57,58], more stress [59] and feel policing stressors differently than men [60,61]. Regarding age, some studies found older police officers presented more stress [62], while others found more stress among young police officers [63]. Police officers with higher educational level used more emotion-focused coping, including seeking support [64].

As demonstrated above, job stress, burnout and coping are crucial psychological constructs, which influence police officers’ tasks and their physical/mental health. Thus, researchers must study these constructs and their relationship, but using questionnaires sensitive to policing tasks idiosyncrasies. This study aims to identify operational and organizational stress, burnout, resilient coping and coping strategies among police officers, as well as to analyze psychometric properties of a Portuguese version of the Organizational Police Stress Questionnaire.

## 2. Materials and Methods

### 2.1. Study Design and Participants

This study is part of a larger cross-sectional project aiming to adapt questionnaires sensitive to Portuguese police tasks. The sample was composed of 1131 police officers of the Portuguese National Police (Polícia de Segurança Pública, PSP), a police force working only in urban areas of Portuguese cities. All districts and Azores/Madeira islands were represented, despite Lisbon and Porto having a larger contribution, reflecting the national distribution of this police force. The sample represents approximately 5% of this force, which is representative for a confidence level of 95% and 3% margin of error (the needed sample was 1020 participants). The participants’ age varied between 20 and 63 years old (M = 42.30; SD = 8.713), with 52.4% having between 20 and 43 years and 47.6% between 44 and 63 years. Job experience in the Portuguese National Police varied between 1 and 40 years (M = 19.12; SD = 8.982), with 51% of the sample having between 1 and 20 years and 49% between 21 and 40 years. Regarding gender, 91% were men, and 9% were women, while in this police force, women are nearly 10%. To avoid the possible identification of individuals from the matching of age, gender and city, no statistical analyses were performed combining these data, and no other sociodemographic data were collected.

### 2.2. Data Collection

This study was approved and carried out in accordance with the authorization/ethic recommendations of the Directorate of the Portuguese National Police (Process 1479-4F05), with online informed consent from all participants. An online questionnaire was prepared on Google Forms, with a link inviting participation in a study of burnout and occupational stress among police officers. The Directorate disseminated this link to police officers using their professional email addresses, and data was collected between September and November 2019. There was no direct contact between participants and researchers, no exclusion criteria existed, and participation was voluntary. The participation rate was nearly 15% of the number of police officers in this police force, and researchers were unable to identify how many police officers read the email, followed the link and decided not to participate. Before responding to the questionnaire, participants were asked to provide their informed consent, with the notification that data would be gathered anonymously. Data were accessed by one researcher only, who downloaded the Excel file, converted it to SPSS format, confirmed the anonymity and created a code for each questionnaire to allow separate studies inside the project.

### 2.3. Questionnaires

The questionnaire was composed of five major groups of questions, and the first group allowed to characterize sociodemographic data (age, sex, job experience and district). The second group was composed of the Operational Police Stress Questionnaire, developed by McCreary and Thompson [10,65] to assess the specificities of job stress among police officers, both for operational and organizational stress sources (PSQ-Op and PSQ-Org). This questionnaire is free for non-commercial, educational and research purposes. After the adaptation of the PSQ-Op [4], this study used only the organizational stress sources. The PSQ-Org questionnaire is composed of 20 items evaluated on a 7-point scale ranging from 1 (“not at all stressful” or “no stress at all”) to 7 (“very stressful” or “a lot of stress”), with 4 indicating moderate stress. In 2017, McCreary and colleagues [66] established norms and cut-off values, with values below 2.0 indicating low stress, between 2.1 and 3.4 indicating moderate stress and above 3.5 indicating high stress. The same procedure was used to create the Portuguese version of the PSQ-Op and PSQ-Org, since it was not published in Portugal. Thus, two psychologists (one conducting research about policing and police forces, another working with police officers) translated the questionnaire into Portuguese. Another researcher, unfamiliar with police officers’ work, subsequently back translated the questionnaire into English and compared it with the original version. Finally, these three researchers discussed each item with two police officers (a patrol police officer and a police station commander) until a lexical and cultural consensus was obtained. Suggestions from police officers were included to add some examples of the Portuguese reality (Table 1). A pilot study was performed with 20 police officers to ensure the questionnaire was easy to complete and was appropriate to the Portuguese situation, and no major changes were made.

The third group of questions was composed by the Spanish Burnout Inventory (SBI, [67]), with a Portuguese version tested on police officers from the Portuguese National Police called PSP, with adequate psychometric properties [68]. The SBI considers burnout as a process of cognitive and emotional deterioration, involving attitudes of indifference and guilt [69], and includes 20 items organized in four scales. The first is enthusiasm towards the job (demonstrating for instance the ambition to accomplish a person’s professional goals because they are a source of personal achievement), while the second is psychological exhaustion (emotional and physical exhaustion related to job tasks, increased by dealing every day with people who present difficulties or problems). The third is indolence (negative attitudes of indifference and cynicism when dealing with people demanding help), while the fourth is guilt (negative feelings, behaviors and attitudes in the workplace, elicited by interactions during labor relations). Each item is assessed by a 5-point frequency scale ranging from 0 (never) to 4 (very frequent or every day). Low scores on enthusiasm towards the job, along with high scores on psychological exhaustion, indolence and guilt, represent high levels of burnout. Scores for each of the four scales are calculated using the mean of the items that compose each scale, and a global score for burnout can be calculated after reversing the items of the enthusiasm scale. Moreover, guilt allows to identify two profiles: Profile 1 (high burnout and low guilt according to cut-off values by Gil-Monte [67] and Profile 2 (high burnout and high guilt). According to Poletto and colleagues [70], it is possible to use percentile analysis to identify burnout at very low levels (P ≤ 10), low levels (11 < P ≤ 33), moderate levels (34 < P ≤ 66), high levels (67 < P ≤ 89) and critical levels (P ≥ 90).

The fourth group of questions was composed of the Brief Resilient Coping Scale [39] in its Portuguese version [38]. The BRCS is composed of four items measuring the person’ capacity to cope with stress in an adaptive way, assessed by a 5-point frequency scale ranging from 1 (almost never) to 5 (almost always). The final score is the sum of the four items, and a score between 1 and 12 represents low resilience, between 13 and 17 moderate resilience, while between 18 and 20 represents high resilience.

The fifth and last group of questions included the Coping Inventory for Stressful Situations (CISS-21, [71]), in its Portuguese version [72], composed by 21 items that measure the use of coping strategies in a stressful situation, namely, task-orientated coping (executing an action to solve the problem at hand, such as “Focus on the problem and try to solve it”), emotion-orientated coping (expressing or feeling emotions, such as “Become very upset”) and avoidance-orientated coping (doing another thing to reduce stress, such as “Treat myself to a snack”). Items are assessed in a 5-point frequency scale ranging from 1 (not at all) to 5 (very much), and each of the three dimensions are calculated from the average of the corresponding 7 items that represent each strategy.

### 2.4. Statistical Analysis

One rule of thumb regarding sample size to perform a factory analysis is that the subject to item ratio should be at least 10 to 1 [73]. Based on this, the sample size was enough to ensure stability of a factor solution and, prior to testing factor structure of the PSQ-Org, data screening for normality and multicollinearity occurred. Then, sample was randomly divided in two, so that mutually independent samples were obtained for the Principal Components Analysis (PCA) and Confirmatory Factor Analysis (CFA). Only observations without any missing items were used, resulting in 1131 observations in total, 339 for the PCA and 792 for the CFA. PCA with oblique rotation was conducted (Promax) to allow for expected factor correlations. For the CFA, the maximum likelihood estimation (ML) with bootstrapping (1000 resamples) was used to generate accurate estimations of standard errors with accompanying confidence intervals (bias-corrected at the 95% confidence level). Three models were estimated: (i) a two-factor model based on the results of the PCA, (ii) a one-factor model representing a general undifferentiated “organizational police stress” latent construct, and (iii) an alternative second-order factor model. These models were compared based on examination of indicators of goodness of fit. As recommended by Kline [74], the model chi-square test (χ^2^) was assessed with a non-significant χ^2^ indicating a good fit. Three approximate fit indexes (RMSEA, CFI and SRMR) were also reported. Values of CFI ≥ 0.90 and RMSEA ≤ 0.10 [75] and SRMR ≤ 0.08 [76] are interpreted as acceptable fit. Reliability was examined with estimates of internal consistency (Cronbach’s alpha coefficient) and Composite Reliability (CR).

To analyze the constructs’ convergent and discriminant validity of the PSQ-Org factors, the average variance extracted (AVE) was estimated. Values of AVE ≥ 0.50 and AVE ≥ r^2^;DV (squared correlation between the factors) were considered indicative of convergent and discriminant validity, respectively [77].

Finally, Pearson’s correlation coefficients were used to determinate whether scales with similar constructs (e.g., operational stress, burnout and resilient coping) were highly correlated with scores from the PSQ-Org, and descriptive analyses were also performed to identify stress/burnout/coping levels.

Data were analyzed using Statistical Package for Social Sciences (SPSS) and Analysis of Moment Structures (AMOS), both version 24 (IBM SPSS/AMOS Inc., Chicago, IL, USA).

## 3. Results

Prior to identifying job stress/burnout/coping level and its relationships, psychometric properties of the PSQ-Org were analyzed, as well its relationship with other constructs assessed in this study. Some detailed statistical analyses were calculated and included as an addendum to the article for those interested (see Supplementary Materials).

### 3.1. Preliminary Analysis

Data screening prior to analysis showed that all inter-correlations were below 0.80 suggesting no multicollinearity [77] (see Supplementary Materials Table S1). An assessment of normality revealed that kurtosis and skewness scores for each item fell within −2 and 2 [78]. The corrected item total correlations were all positive and more than 0.40. The internal consistency of the total scale was good (α = 0.95), and there was a low variation in Cronbach’s alpha if items were deleted. Six items (1, 2, 14, 15, 17 and 19) were excluded from the original scale in Bangladeshi culture [79]. A four-factor model for the PSQ-Org was obtained, consisting of 14 items. In this study, it was not possible to confirm this factorial structure, as all items had adequate properties in this preliminary analysis.

#### 3.1.1. Principal Components Analysis

PCA suggested that a two-factor structure explained 60% (59.67) of the variance. The Kaiser–Meyer–Olkin (KMO) Measure was 0.95, indicating that the sample was adequate, and Bartlett’s Test of Sphericity was significant (χ^2^; = 4661.02, *p* < 0.001). A Scree plot of the eigenvalues was considered (see Supplementary Materials Figure S1), also indicating a two-factor solution. The first factor was robust, with a high eigenvalue of 10.6, and it accounted for 52.93% of the variance in the data. The eigenvalue for Factor 2 was 1.35, accounting for 6.74% of the total variance. Factor 1 comprised items 2, 4, 5–7, 9, 12–14, 16 and 20 (11 items), which we termed as “Poor management and lack of resources”. Factor 2 included items 1, 3, 8, 10, 11, 15 and 17–19 (9 items), which we designated as “Responsibilities and burden”. Table 2 shows the factor loadings, and item 10 presented cross loading. However, we decided to keep this item due to its properties (e.g., corrected item-total correlation of 0.72). We consider that item 10 fitted conceptually to Factor 2. All items had communalities greater than 0.40 [80]. Internal consistencies for each of the two subscales were good (see Table 2).

#### 3.1.2. Confirmatory Factor Analysis: Alternative Models

As the PCA suggested a two-factor solution, a confirmatory factor analysis (CFA) was used to examine this structural model. We considered 792 observations in total for the CFA. Mardia’s kurtosis coefficient of 177.90 with a critical ratio of 84.39 indicated that the data were multivariate non-normal and may result in standard error biases [81]. Accordingly, analysis used ML estimation with bootstrapping showing a poor fit (χ^2^(169) = 1789.82, *p* < 0.001; CFI = 0.84; RMSEA = 0.11 (90% CI 0.11–0.12); SRMR = 0.06). However, all standardized factor loadings were generally large and statistically significant for two factors (0.67–0.79 and 0.57–0.80 for factors 1 (“Poor management and lack of resources”) and 2 (“Responsibilities and burden”), respectively).

The two factors were correlated substantially with each other (r = 0.871). The average variance extracted (AVE), with values of 0.53 for Factor 1 and 0.50 for Factor 2, indicated satisfactory convergent validity. However, the AVE values were smaller than the squared correlation between the factors (r^2^DV = 0.76), which led us to test the fit of a single factor structure. This one-factor model did not fit the data well statistically nor descriptively (χ^2^(170) = 2133.68, *p* < 0.001; CFI = 0.81; RMSEA = 0.12 [90% CI 0.12–0.13]; SRMR = 0.07). Comparing the two factorial structures, the difference from models was statistically significant (χ^2^(1) = 343.86, *p*< 0.001) indicating that proposed framework by the PCA showed a significantly better fit to the theoretical model. Furthermore, more than 50% of the items presented high error covariance.

**Figure 1:**
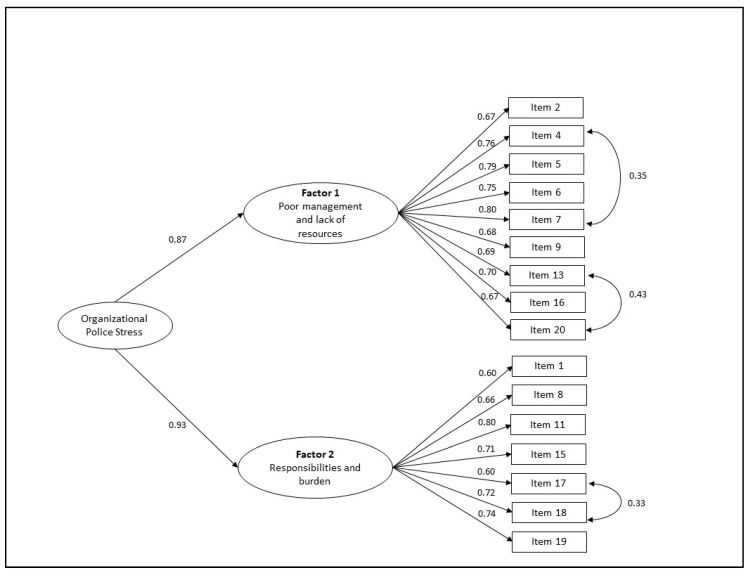
Second-order model for the PSQ-Org (Database 2; *n* = 792).

In such circumstances, a second-order factor model was generated and tested (Figure 1). This alternative solution examined whether a latent general reality testing factor existed (“Organizational police stress”, such as McCreary and Thompson [15,65,66] considered when they developed the instrument and also other authors who adapted it for different countries and realities [12,79,82,83]), in addition to two dimensions (“Poor management and lack of resources” and “Responsibilities and burden”, where all items are related with organizational aspects). Based on high modification indices, the two first order dimensions have been re-specified. Items 3, 10, 12 and 14 loaded in more than one factor and, therefore, were excluded in this analysis. We also allowed errors to covary for items 4 and 7, 13 and 20, and 17 and 18. After estimating the model, we verified that this model did not fit well statistically (χ^2^(100) = 851.93, *p* < 0.001), but it did fit well descriptively (CFI = 0.90; RMSEA = 0.10 [90% CI 0.09–0.10]; SRMR = 0.06). It should be noted that the χ^2^; value is sensitive to sample size, indicating significant misfit even in good fit models [84]. All factor loadings were statistically significant (Figure 1). This 16-item solution should be preferred overall. The reliability of the two subscales was good (α= 0.91 and CR = 0.91 for Factor 1 and α = 0.88 and CR = 0.87 for Factor 2). Good internal consistency was obtained for the full scale involving 16 items (α = 0.93).

### 3.2. Job Stress, Burnout and Coping among Police Officers: Descriptive Statistics and Group Differences

Considering instruments’ cut-off values (Table 3), findings showed operational stress is high for 88.4% of the sample, while organizational stress is high for 87.2%. Regarding burnout, for Enthusiasm 9.8% of the sample presented a very low level and 23.7% a low level. The percentage of participants who presented high and critical levels was, respectively, 22.6% and 12.2% for Psychological exhaustion, 26.9% and 10.2% for Indolence, 35.3% and 10.3% for Guilt and 23.7% and 10.9% for Burnout. With Gil-Monte’s profiles [67], 35% of the sample (*n* = 396) was included in Profile 1 (high burnout but low guilt), and only 9.5% (*n* = 108) was included in Profile 2 (high burnout and guilt), while the rest of the sample did not present any specific profile. Finally, regarding resilient coping 53.8% of the sample presented low values, while 24.7% presented moderate values, and only 11.5% presented a high resilient coping.

Descriptive analyses for all dimensions were performed (Table 4) and showed at least one participant had the minimum or the maximum value allowed by the scales’ range. Standard deviation was higher for the responsibilities and burden dimension of organizational stress as well for the psychological exhaustion dimension of burnout. Considering mean values, for Operational stress, levels were high and higher than Organizational stress. Poor management and lack of resources presented the highest value for job stress, while Responsibilities and burden had the lowest value for job stress. Burnout presented moderate mean values, higher for Psychologic exhaustion and Indolence, than for Enthusiasm and Burnout, while Guilt presented a low value. Regarding coping, resilient coping presented moderate levels, while task orientated presented the highest value, followed by emotion-coping and avoidance orientated coping, which presented the lowest value.

Comparative analyses were also performed regarding gender (Table 4) using non-parametric tests due to the different proportions of men and women (which reflects the characteristics of this population at national level), despite Student’s t-tests showing the same significant results. Women presented higher enthusiasm towards the job and avoidance-orientated coping, while men presented higher indolence and burnout. Regarding age and job experience, a detailed analysis was performed comparing groups according to adulthood stages/responsibilities and job experience phases related with professional progression in this police force (see Supplementary Materials Table S2). This analysis showed older police officers with less operational and organizational stress, less indolence and more guilt and emotion-focused coping than younger police officers. Resilient coping seemed to decrease in the group of middle adulthood and increase on older police officers, while avoidance-orientated coping is typical of younger adults, decreasing in middle age and older adults. Considering job experience, a similar pattern was found, with more experienced police officers presenting less operational stress, less insolence, less avoidance-orientated coping and more guilt and emotion-focused coping than those with less experience. Resilient coping seemed to decrease between 1 and 30 years of job experience, increasing for the group of 31 to 40 years of job experience. Additionally, correlational analysis (see Supplementary Materials Table S2) showed that older police officers and those with more job experience presented more guilt and more emotion-orientated coping, while younger professionals and with less experience presented more operational stress, indolence and avoidance-orientated coping. Only job experience had a negative correlation with organizational stress, while enthusiasm towards the job, psychological exhaustion, burnout, resilient coping and task-orientated coping appear not be influenced by age nor by job experience.

### 3.3. Job Stress, Burnout and Coping’s Relationships

To understand the relationship between job stress, burnout and coping, R Pearson’s correlations were performed (Table 5), contributing also for convergent validity evidence of the PSQ-org questionnaire. Overall, we observed moderate to strong correlations in the expected direction between 16-item PSQ-Org scale score and its two dimensions, being related conceptually with distinct constructs: operational stress and burnout, as well with coping. Scores in organizational stress dimensions were positively associated with operational stress, psychological exhaustion, indolence and, less strongly, guilt. In turn, enthusiasm subscale showed a negative correlation with organizational stress. Furthermore, we found low to moderate correlations between total scores and subscales of organizational stress and resilient coping. Interestingly, higher scores in resilient coping seem to indicate lower organizational stress. Task-oriented coping was more strongly associated with the dimension related to responsibilities and burden than with the dimension related to poor management and lack of resources. These patterns support the construct validity of the scale. It was also found that operational stress correlated positively with burnout’s dimensions (except for enthusiasm towards job, with a negative correlation) and with emotion and avoidance orientated coping and correlated negatively with resilient and task-orientated coping. Regarding burnout’s dimensions, enthusiasm towards job correlated positively with resilient and task orientated coping and negatively with emotion-orientated coping, while for avoidance orientated coping no significant relationship was found. Psychological exhaustion, indolence, guilt and burnout presented the same pattern, correlating negatively with resilient and task orientated coping and positively with emotion and avoidance orientated coping. Finally, resilient coping correlated negatively with emotion-orientated, and positively with task and avoidance coping.

## 4. Discussion

To identify operational and organizational stress, burnout, resilient coping and coping strategies among police officers, it is important to resort to instruments that reflect policing idiosyncrasies. The validation of a Portuguese version of the Organizational Police Stress Questionnaire found adequate psychometric proprieties, despite deleting four items. These items can reflect characteristics of this study’s sample, which was formed from a national police force working in major Portuguese cities, or can reflect the lack of expression of the organization’s specific rules in these items (e.g., items “10. Perceived pressure to volunteer free time” and “14. Unequal sharing of work responsibilities”), cultural/individual style (e.g., “3. Feeling like you always have to prove yourself to the organization”) or even a question about hierarchy (e.g., “12. Inconsistent leadership style”) that may have elicited fear to be identified, despite the guarantee of anonymity and confidentiality. Results also revealed a two-factor structure, “Poor management and lack of resources” and “Responsibilities and burden”, confirming the existence of organizational stressors and confirming PSQ-Org as a valid and reliable measure of policing job stress, such as the one McCreary and Thompson [15,65,66] referred to when creating this instrument.

Other studies found different structures and factors, namely, because organizational stress is highly dependent and related to each police force’s cultural idiosyncrasies. For instance, Shane [12], with 461 USA police officers, suggested 19 from the 20 original items, organized in six factors: Co-worker relations, Training and resources, Leadership and supervision, Bureaucracy, Internal affairs and Accountability, and Management and organizational capacity (with only two items). Sagar et al. [79], with 210 police officers from Bangladesh suggested 14 from the original 20 items, organized in four factors, namely Poor management and bureaucracy, Lack of manpower and resources, Feelings of excessive duty and being supervised, and Lack of leisure time and negative evaluations. Irniza et al. [82], with 262 police officers, adapted for Malay the PSQ, both operational and organizational scales. They excluded four factors related with lifestyle (managing social life outside work, eating healthy at work, making friends outside the job and finding time to stay in good physical condition), suggesting the rest of the items loaded in one dimension including operational and organizational stressors. More recently, Kaplan et al. [83], during an 8-week resilience training course, studied 72 USA police officers and found a good internal consistency for the PSQ, for both operational (α = 0.85) and organizational (α = 0.88) scales. Like Irniza et al. [82] defended, PSQ-Org diverse factor structures can result from the cross-cultural specificities of each country. However, these differences are less evident for the PSQ operational scale, which can reflect more common police stressors all over the world. Moreover, the high standard deviation values for organizational stress, despite the scale ranged from 1 to 7 points, can also reflect different organizational management of each department/police station.

Regarding job stress, burnout and coping and despite mean values showing moderate to high levels, cut-off scores suggested a more worrying reality, with 88.4% of the sample presenting high operational stress and 87.2% high organizational stress levels. For burnout, 23.7% and 10.9% of police officers presented high and critical levels, respectively. Moreover, critical levels were also found in 12.2% for Psychological exhaustion, 10.2% for Indolence and 10.3% for Guilt, while 9.5% presented for Guilt the Profile 2, which is characterized by high burnout and guilt levels, suggesting psychological suffering. Regarding resilient coping, 53.8% of the sample presented low resilience, while task-orientated coping was higher than emotion and avoidance orientated coping. Resilient coping correlated negatively with emotion-orientated and positively with task and avoidance coping, suggesting avoidance strategies are sometimes used for managing stress [44,69]. These results are in line with other studies and confirm that policing is a stressful professional occupation [1,2,15,28] and that burnout affects police officers [4,6,24,27]. Moreover, results confirmed the preference of task-orientated coping among police officers [41,43,44,45], despite low values of resilience coping. However, as a descriptive study, no causal relationships can be inferred from correlational analysis, and it is important to continue to study these phenomena.

The influence of individual factors such as gender, age and job experience revealed some different patterns on the psychological variables assessed. In line with other studies, women are a minority in police forces [56], but in this study, they presented higher enthusiasm towards the job and avoidance-orientated coping, while men presented higher indolence and burnout. These results confirm studies showing men presented more burnout [85] but differ from other studies which showed women experienced more emotional exhaustion, less depersonalization and more stress [52,57,58,59], preferring to use emotion-focused strategies [53]. A possible explanation can be women are entering into nontraditional occupations where they have been excluded or marginalized for cultural/social or discrimination reasons [86] and are nowadays proud to access these jobs, policing career included. Conceptually, avoidance-orientated coping is a strategy of emotion-focused coping, and in our sample, this preference by women can reflect that women police officers feel policing stressors differently than men [60,61]. Moreover, avoidance coping can be useful to alleviate stress when the person does not have enough resources to solve the problem directly [37]. Regarding age and job experience, results are in line with other studies, revealing that older police officers and those with more years of job experience presented less operational and organizational stress, less burnout dimensions [63,87] and more resilience [54]. These results can reflect that while suffering from burnout, the workers tend to leave their occupations, and only the healthier ones remain on the job, a phenomenon known as the “healthy worker effect” [88]. Finally, older/with more job experience police officers felt more guilty and used more emotion-focused coping while younger/with less job experience felt less guilty and preferred avoidance-orientated coping. Guilt, as other negative emotions, can be a psychological consequence of critical incidents or moral injury, frequent situations for police officers [89,90]. Avoidance coping used by younger police officers can reflect their lack of resources to eliminate the stressor directly [37], while emotion-focused coping used by older officers can reveal they learned how to cope with stressors that cannot be changed.

Considering all results, it is important to implement programs to increase resilience [91,92], to manage stress [44,85,93] and to promote the use of adequate coping strategies [27,42,51,94] but adapted to individual differences such as gender, age and job experience, since they can affect the way a police officer faces job stressors. In the future, job stress management, burnout prevention and coping training are important topics to be studied. In addition, it is important to identify which coping strategies are mostly used during critical incidents and how post-traumatic stress is presented, as well as substance use/abuse as a coping strategy, like other studies did [48,95]. Moreover, the significant correlations between these constructs suggest they are associated and influence behavior and emotional states, which alerts to the need to study them associated with internal and external aggression, a current concern for researchers [7,8,13,29,30,32,33]. Despite not being analyzed in this study, Patterson [64] found police officers with higher educational level used more emotion-focused coping, including seeking support, an interesting result that demands further analyses. In fact, in stress management programs, social/emotional seeking support should be emphasized [94], overtaking the frequent public image of “cops don’t cry” [96] and helping to regulate emotion suppression [97] and changing social perceptions of police officers as Robocops [27].

## 5. Conclusions

This study found the Portuguese version of the Organizational Police Stress Questionnaire to be a valid and reliable measure of policing job stress, with two factors (Poor management and lack of resources, Responsibilities and burden). Using this instrument and others to assess burnout and coping, this study identified high levels of job stress and burnout and low resilient coping, as well as the preference for task-orientated coping, with some differences across gender, age and job experience. The relationships between job stress, burnout and coping suggested these constructs need to be considered as affecting policing tasks and police officers’ mental health, since stressful situations imply the use of both individual and organizational resources. However, the study has some limitations, namely, cross-sectional and descriptive study based only in voluntary participation from one of the three major Portuguese Police forces; the sample represented only 5% of the national police force and had 3% of margin of error; not having studied more sociodemographic or labor factors on psychological variables possible bias from the healthy worker effect since we do not have information on the participants’ psychological health status, namely, if they are working while ill or have returned to work after being ill; possible bias from socially desirable responses, which we tried to control by previously informing participants that the questionnaire was anonymous and confidential, and that results will only be accessed by researchers and not by the police force; lack of control to identify participants who accessed the questionnaire and give up its submission; and the timing of data collection, which did not reflected current stressors such as the COVID-19 pandemic. All these limitations demand more studies on these topics, while trying to collect data from larger samples and to use samples from other national police forces, namely, judiciary and militarized ones, and thus analyze with more detail the specific operational and organizational stressors of each police force. Moreover, in the future, it will be important to collect data combining burnout, resilience and coping among other occupational groups and compare it with that of different police forces.

Nowadays, police officers are often exposed to high-risk and high-stress events, caused by nature (e.g., natural disasters) or by human action (e.g., terrorists attacks, multi-victims accidents), alerting to the need to measure the burden of psychological impact from the involvement in critical incidents [98]. Results showed operational and organizational stressors are high, and currently, with the COVID-19 pandemic, police forces face new risks and challenges, which require organizations to reinforce their resilience resources with training for facing these new situations [99]. Moreover, social support and coping must be considered as useful resources, as well as mental health support [98]. Thus, new directions must be considered in police training, preparing police officers “to meet the contemporary challenges of police work” [100] (p. 1) but also to develop stress/burnout prevention/management programs, as well as to promote resilience and adequate coping strategies, including emotional intelligence [101,102,103]. As Sinclair et al. [104] (p. 1) recently noted, now more than ever, “occupational health science in time of COVID-19” is crucial to help all workers, including police officers, whose professional occupation is one of the most stressful. With this study, we contribute with the adaptation of a specific questionnaire to measure job stress among police forces but also bring attention to the importance of psychological variables and the benefits of promoting psychological/occupational health in police forces, so as to reduce burnout and job stress.

## Figures and Tables

**Table 1:** Organizational police stress questionnaire (PSQ-Org) original and Portuguese versions.

Original PSQ-Org (McCreary and Thompson (2006)	Portuguese Version of PSQ-Org				
No Stress at All	Moderate Stress	A Lot of Stress	Nenhum Stress	Stress Moderado	Muito Stress
1	4	7	1	4	7
1. Dealing with co-workers	1.Ter de lidar com colegas de trabalho.				
2. The feeling that different rules apply to different people (e.g., favoritism)	2. Sentir que diferentes regras são aplicadas a diferentes pessoas (ex.: favoritismo).				
3. Feeling like you always have to prove yourself to the organization	* 3. Sentir que tem de estar sempre a provar o seu valor à instituição.				
4. Excessive administrative duties	4. Existir excesso de tarefas administrativas.				
5. Constant changes in policy/legislation	5. Existirem mudanças constantes nas políticas e legislação.				
6. Staff shortages	6. Redução do número de polícias disponíveis.				
7. Bureaucratic red tape	7. Existir demasiada burocracia.				
8. Too much computer work	8. Ter que trabalhar demasiado no computador.				
9. Lack of training on new equipment	9. Falta de treino com novos equipamentos.				
10. Perceived pressure to volunteer free time	* 10. Pressão para se voluntariar ou estar disponível mesmo nos tempos livres.				
11. Dealing with supervisors	11. Lidar com superiores/comandantes.				
12. Inconsistent leadership style	* 12. Estilo de liderança/chefia/comando inconsistente.				
13. Lack of resources	13. Falta de recursos.				
14. Unequal sharing of work responsibilities	* 14. Partilha desigual de responsabilidades profissionais.				
15. If you are sick or injured your co-workers seem to look down on you	15. Sentir que quando está doente ou lesionado os colegas parecem olhá-lo de outra forma.				
16. Leaders over-emphasize the negatives (e.g., supervisor evaluations, public complaints)	16. Os superiores valorizarem mais os aspetos negativos da profissão (ex.: queixas do cidadão, avaliações de superiores/chefias/comandantes).				
17. Internal investigations	17. Lidar com investigações internas.				
18. Dealing the court system	18. Lidar com o sistema judicial.				
19. The need to be accountable for doing your job	19. Necessidade de ser responsabilizado pelo seu trabalho.				
20. Inadequate equipment	20. Usar equipamento inadequado.				
The Operational Police Stress Questionnaire is provided free for non-commercial, educational, and research purposes. Cite as: - McCreary, D.R., and Thompson, M.M. (2013). The Operational Police Stress Questionnaire (PSQ-Org). Measurement Instrument Database for the Social Science. Retrieved from www.midss.ie	O Questionário de Stress Operacional é de acesso livre para efeitos de uso não comercial, educacional e investigação. Citar como: - versão original de: McCreary, D.R., & Thompson, M.M. (2013). The Operational Police Stress Questionnaire (PSQ-Org). Measurement Instrument Database for the Social Science. Disponível em www.midss.ie (https://www.midss.org/sites/default/files/psq-org.pdf) - versão portuguesa de: Queirós, C., Passos, F., Bártolo, A., Faria, S., Fonseca, S.M., Marques, A.J., Fernandes da Silva, C., & Pereira A. (2020). Job stress, burnout and coping in police officers: relationships and psychometric properties of the Organizational Police Stress Questionnaire; *International Journal of Environmental Research and Public Health*, *17*, 6718; doi:10.3390/ijerph17186718				

Note: * Items with poor psychometric properties, suggesting a Portuguese version with only 16 items.

**Table 2:** Factor Analysis of the PSQ-OR (20 items): two-factor solution using a part of the sample (Database 1; *n* = 339).

	Factor 1	Factor 2	
Item (Item No.)	Poor Management and Lack of Resources (α = 0.927)	Responsibilities and Burden (α = 0.904)	*h^2^;*
1		0.881	0.475
2	0.414		0.514
3		0.591	0.628
4	0.594		0.572
5	0.545		0.627
6	0.929		0.665
7	0.813		0.693
8		0.544	0.426
9	0.539		0.521
10	(0.441)	0.409	0.620
11		0.845	0.679
12	0.442		0.550
13	1062 *		0.737
14	0.650		0.641
15		0.796	0.504
16	0.445		0.542
17		0.711	0.601
18		0.616	0.647
19		0.735	0.651
20	0.863		0.641

α = Cronbach’s alpha coefficients; *h*^2^ = Communalities; Factor loadings < 0.40 were suppressed (based on Hair et al. [77]). Note: * if the factors are correlated (oblique), the factor loadings are regression coefficients and not correlations and as such, they can be larger than one in magnitude.

**Table 3:** Sample’s frequency (and percentage) distribution according established cut-off points.

Questionnaires’ Scales	Established Levels with Cut-Off Points				
PSQ	Low stress (≤2)		Moderate stress (2.1–3.4)		High stress (≥3.5)
Operational stress	29 (2.6)		102 (9.0)		1000 (88.4)
Organiz. stress	26 (2.3)		119 (10.5)		986 (87.2)
SBI	Very low level (P ≤ 10%)	Low (P11–33)	Moderate (P34–66)	High (P67–89)	Critical level (P ≥ 90)
Enthusiasm job	111 (9.8)	268 (23.7)	349 (30.9)	269 (23.8)	134 (11.8)
Psychol. exhaustion	111 (9.8)	265 (23.4)	361 (31.9)	256 (22.6)	138 (12.2)
Indolence	81 (8.0)	221 (19.5)	400 (35.4)	304 (26.9)	115 (10.2)
Guilt	206 (18.2)	213 (18.8)	196 (17.3)	399 (35.3)	117 (10.3)
Burnout	123 (10.9)	245 (21.7)	372 (32.9)	268 (23.7)	123 (10.9)
BRCS	Low resilience (4–12)		Moderate resilience (13–17)		High resilience (18–20)
Resilient coping	609 (53.8)		392 (34.7)		130 (11.5)

**Table 4:** Descriptive and comparative statistics for job stress, burnout and coping.

Scales (Range)	Mean	Std. Deviation	Men *n* = 1029	Women *n* = 102	U
Operational stress (1 to 7)	5.026	1.291	5.034	4.942	0.323
Poor manag. and lack of resources (F1)	5.520	1.243	5.535	5.375	0.109
Responsibilities and burden (F2)	4.126	1.490	4.125	4.130	0.845
Organizational stress	4.910	1.259	4.918	4.830	0.377
Enthusiasm towards the job (0 to 4)	1.935	0.947	1.913	2.153	0.016 *
Psychological exhaustion	2.366	1.091	2.381	2.218	0.149
Indolence	2.211	0.987	2.246	1.868	0.000 ***
Guilt	0.881	0.796	0,882	0.873	0.898
Burnout	1.873	0.742	1.892	1.684	0.007 **
Resilient coping (1 to 5)	3.122	0.980	3.120	3.137	0.779
Task-orientated coping (1 to 5)	3.574	0.846	3.565	3.668	0.288
Emotion-orientated coping	2.513	0.834	2.501	2.639	0.067
Avoidance-orientated coping	2.506	0.846	2.484	2.723	0.012 *

Note: * *p* ≤ 0.050 ** *p* ≤ 0.010 *** *p* ≤ 0.001; U = Mann–Whitney test.

**Table 5:** Pearson correlations between Organizational police stress, Operational police stress, Burnout and Coping (*n* = 1131).

Dimensions	Organizational Stress	Factor 1	Factor 2	Operational Stress	Burnout	Enthusiasm Tow. Job	Psychological Exhaustion	Indolence	Guilt	Resilient Coping
Operational stress	0.795	0.766	0.714							
Burnout	0.683	0.599	0.675	0.630						
Enthusiasm towards the job	−0.404	−0.358	−0.397	−0.399						
Psych. exhaustion	0.685	0.620	0.658	0.645						
Indolence	0.669	0.615	0.631	0.609						
Guilt	0.318	0.213	0.386	0.262						
Resilient Coping	−0.263	−0.184	−0.310	−0.257	−0.432	0.428	−0.339	−0.307	−0.272	
Task-oriented	−0.207	−0.121	−0.269	−0.183	−0.330	0.337	−0.241	−0.229	−0.226	0.571
Emotion-oriented	0.416	0.338	0.440	0.395	0.521	−0.257	0.499	0.420	0.464	−0.320
Avoidance-oriented	0.171	0.167	0.152	0.163	0.077	0.023 *	0.091	0.086	0.085	0.148

Note: All correlations were significant at *p* < 0.001 * non-significant correlation.

## Supplementary Materials

The datasets generated for this study are available on request to the corresponding author, after authorization from the National Portuguese Police. The following are available online at https://www.mdpi.com/1660-4601/17/18/6718/s1. Figure S1: Scree Plot in favor of the two-factor structure. Table S1: PSQ-Org items descriptive statistics (*n* = 1131). Table S2: Comparative analysis according to age stages and job experience phases and correlational analysis for job stress, burnout and coping.
