# Supplementary material for: Job Stress, Burnout and Coping in Police Officers: Relationships and Psychometric Properties of the Organizational Police Stress Questionnaire
Source: Int J Environ Res Public Health. 2020 Sep 15;17(18):6718. doi: 10.3390/ijerph17186718 (PMC7557776; doi:10.3390/ijerph17186718)
Supplement: Supplementary file 1 [file ijerph-17-06718-s001.pdf]

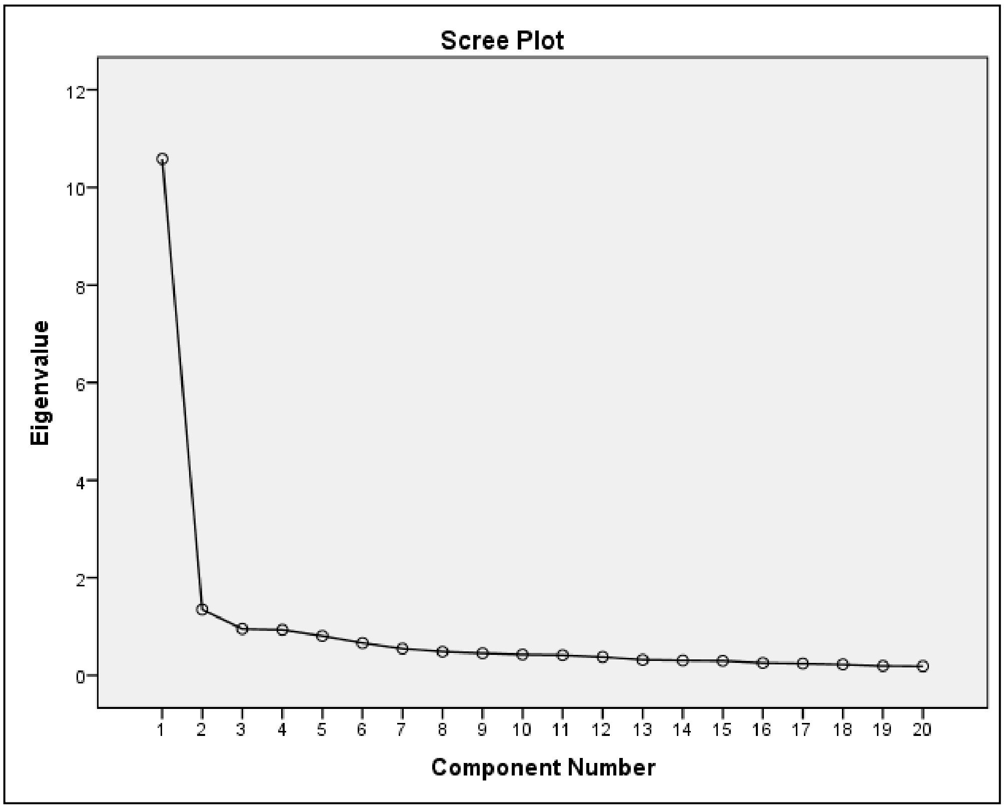

Figure S1. Scree plot in favor of the two-factor structure.

Table S1. PSQ-Org items descriptive statistics (*n* = 1131).

Item	M	SD	Skewness	Kurtosis	1	2	3	4	5	6	7	8	9	10	11	12	13	14	15	16	17	18	19	20
**1**	3.23	1.72	0.403	−0.612	_	0.397	0.422	0.341	0.367	0.233	0.309	0.402	0.278	0.334	0.538	0.389	0.216	0.377	0.472	0.284	0.354	0.366	0.418	0.222
**2**	5.43	1.67	−0.901	−0.067		_	0.601	0.524	0.528	0.460	0.488	0.341	0.418	0.536	0.500	0.609	0.453	0.636	0.399	0.555	0.459	0.449	0.396	0.449
**3**	4.90	1.86	−0.592	−0.713			_	0.627	0.591	0.468	0.538	0.424	0.449	0.582	0.601	0.575	0.425	0.584	0.521	0.557	0.525	0.513	0.538	0.458
**4**	5.19	1.72	−0.664	−0.565				_	0.664	0.536	0.736	0.545	0.488	0.511	0.486	0.502	0.477	0.573	0.430	0.494	0.477	0.512	0.457	0.467
**5**	5.23	1.70	−0.750	−0.362					_	0.596	0.680	0.492	0.527	0.531	0.479	0.514	0.486	0.573	0.435	0.498	0.444	0.552	0.474	0.483
**6**	6.10	1.35	−1.675	2.294						_	0.679	0.375	0.489	0.467	0.388	0.444	0.664	0.514	0.346	0.495	0.378	0.465	0.409	0.544
**7**	5.78	1.52	−1.265	0.826							_	0.517	0.501	0.514	0.469	0.483	0.582	0.575	0.380	0.531	0.445	0.546	0.448	0.511
**8**	4.17	1.95	−0.111	−1.102								_	0.527	0.444	0.520	0.383	0.347	0.453	0.472	0.332	0.424	0.454	0.458	0.316
**9**	4.89	1.83	−0.480	−0.819									_	0.524	0.453	0.475	0.523	0.521	0.421	0.466	0.426	0.502	0.444	0.550
**10**	4.82	1.93	−0.481	−0.910										_	0.626	0.575	0.452	0.586	0.529	0.524	0.495	0.499	0.519	0.505
**11**	4.10	1.97	−0.058	−1.130											_	0.619	0.378	0.582	0.589	0.506	0.545	0.536	0.587	0.410
**12**	5.14	1.78	−0.715	−0.500												_	0.469	0.666	0.451	0.569	0.476	0.488	0.460	0.482
**13**	6.02	1.40	−1.523	1.680													_	0.549	0.337	0.481	0.398	0.479	0.368	0.702
**14**	5.36	1.69	−0.862	−0.163														_	0.475	0.559	0.485	0.516	0.507	0.499
**15**	3.61	2.06	0.276	−1.224															_	0.446	0.468	0.444	0.486	0.358
**16**	5.47	1.80	−1.036	−0.019																_	0.517	0.534	0.473	0.532
**17**	4.52	2.07	−0.321	−1.202																	_	0.683	0.643	0.450
**18**	4.93	1.92	−0.591	−0.836																		_	0.649	0.529
**19**	4.31	2.04	−0.136	−1.221																			_	0.461
**20**	5.57	1.66	−1.022	0.075																				_

Table S2. Comparative analysis according to age stages and job experience phases and correlational analysis for job stress, burnout and coping.

Scales (range)	Age	Job experience	age			p ANOVA post-hoc	Job experience				p ANOVA post-hoc
			20 to 34 n = 246 (a)	35 to 49 n = 620 (b)	50 to 63 n = 265 (c)		1 to 10 n = 238 (a)	11 to 20 n = 338 (b)	21 to 30 n = 450 (c)	31 to 40 n = 103 (d)	
Operational stress (1 to 7)	-,132 **	-,137 **	5.167	5.120	4.675	0.000 *** ab / c	5.184	5.220	4.901	4.591	0.000 *** ab / cd
Poor man. and lack resources (F1)			5.598	5.583	5.301	0.005 ** ab / c	5.634	5.649	5.441	5.206	0.003 ** ab / d
Responsibilities and burden (F2)			4.075	4.211	3.973	0.078	4.134	4.207	4.110	3.929	0.414
Organizational stress	-,057	-,065 *	4.932	4.983	4.720	0.017 * b / c	4.978	5.018	4.859	4.647	0.038 *
Enthusiasm towards the job (0 to 4)	-,015	-,004	2.028	1.869	2.002	0.035 *	1.982	1.895	1.916	2.052	0.404
Psychological exhaustion	,011	,007	2.293	2.439	2.265	0.046 *	2.318	2.433	2.353	2.330	0.597
Indolence	-,098 **	-,104 **	2.306	2.265	1.999	0.000 *** ab / c	2.335	2.305	2.144	1.926	0.001*** ab / d
Guilt	,082 **	,081 **	0.784	0.882	0.968	0.032 * a / c	0.769	0.849	0.948	0.951	.026 * a / c
Burnout	-,009	-,017	1.839	1.920	1.794	0.049 *	1.861	1.916	1.872	1.768	0.354
Resilient coping (1 to 5)	-,058	-,055	3.287	3.042	3.155	0.003 ** a / b	3.272	3.095	3.023	3.306	0.003 ** c / ad
Task-orientated coping (1to 5)	-,033	-,035	3.658	3.508	3.650	0.015 *	3.660	3.520	3.543	3.703	0.078
Emotion-orientated coping	,097 **	,089 **	2.418	2.493	2.649	0.005 ** ab / c	2.398	2.518	2.527	2.714	0.014 * a/ d
Avoidance-orientated coping	-,126 **	-,143 **	2.677	2.462	2.449	0.002 ** a / bc	2.724	2.546	2.351	2.562	0.000 *** ab / c

Note: * p ≤ .050; ** p ≤. 010; *** p ≤. 001; a / b /c / d: Hochberg's GT2 post-hoc test for groups' opposition.
